# Surgical technique to prevent spillage of cyst fluid during operation for cystic ovarian tumors

**DOI:** 10.1007/s00383-013-3277-9

**Published:** 2013-02-09

**Authors:** Eiichiro Watanabe, Kiyoshi Tanaka, Noriko Takeda, Hajime Takayasu, Kazuko Yokota, Masahiko Watanabe

**Affiliations:** Department of Surgery, School of Medicine, Kitasato University, 1-15-1 Kitasato, Minami-ku, Sagamihara, Kanagawa 252-0374 Japan

**Keywords:** Spillage, Ovarian cyst, Surgical sheet, Aron Alpha, S.A.N.D. balloon catheter

## Abstract

We describe a new technique to prevent spillage of cyst fluid in patients undergoing surgery for cystic ovarian tumors. The cyst is first covered with a sterilized surgical sheet applied with quick-drying glue and is then punctured. This technique completely prevents spillage of cyst fluid into abdominal cavity.

## Introduction

Spillage of ovarian cyst fluid during surgery is associated with a number of risks, including chemical peritonitis, gliomatosis peritonei, pseudomyxoma peritonei, tumor recurrence, and dissemination of malignant cells [1–6]. Several reports have described the use of a suction tube such as a S.A.N.D. balloon catheter to prevent spillage of cyst fluid, but the reliability of some procedures has been questioned because the direct puncture of cysts unavoidably results in the release of some cyst fluid. We developed a new technique to completely prevent spillage of cyst fluid. This technique also allows a smaller surgical incision, resulting in good cosmetic results.

## Operative technique

**Fig. 1:**
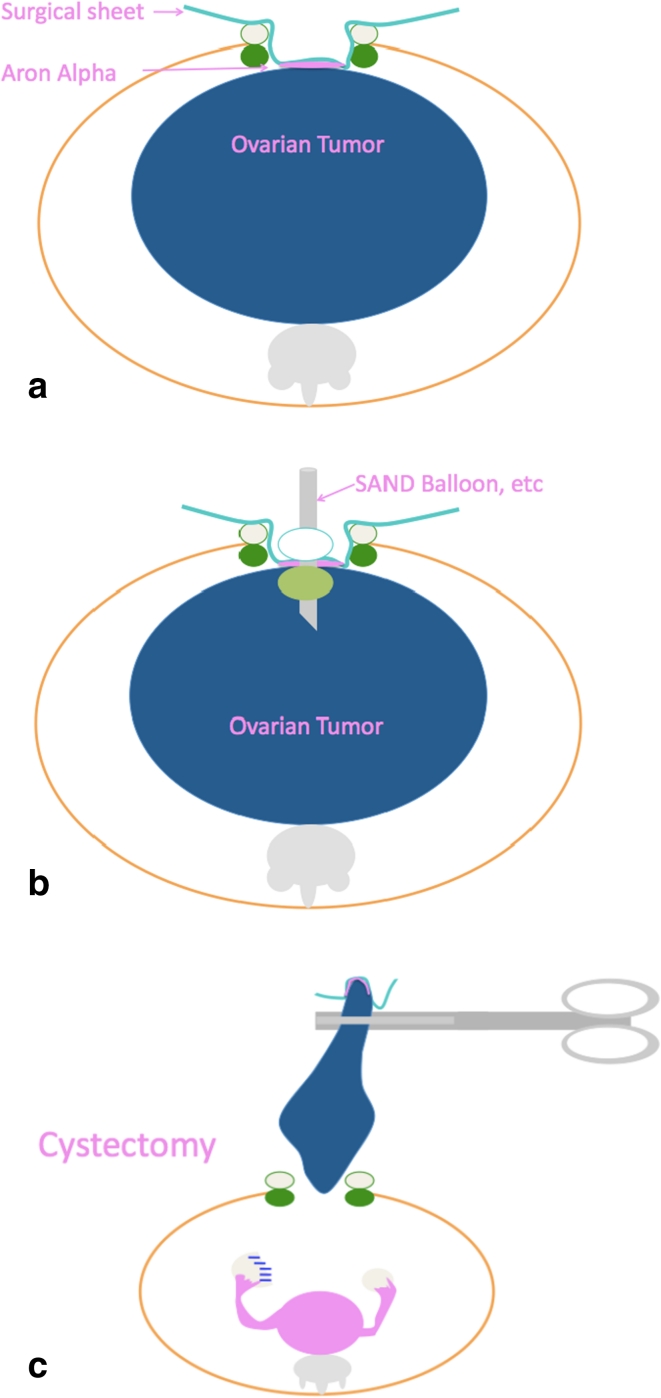
**a** A small Pfannenstiel incision is made, and the surgical field is maintained with the use of a wound retractor (Alexis^®^). After wiping the surface of the tumor located just under the wound with gauze, the rough side of a sterilized surgical sheet (ELS-102S) is attached to the cyst using quick-drying glue (Aron Alpha A <Sankyo>^®^) (Aron Alpha cannot work when applied to the vinyl coating side of the sheet). **b** After the sheet is glued tightly to the tumor, requiring several seconds, the cyst is punctured with a suction tube, such as a S.A.N.D. balloon catheter, and the cyst fluid is aspirated as extensively as possible. **c** The cyst shrinks, and the tumor is then brought outside of the body, while tightly clamping the puncture site to prevent spillage of cyst fluid remaining in the tumor. Finally, an ovarian cystectomy is performed, while safely preserving the healthy ovarian tissue on the affected side

With the patient under general anesthesia, a small Pfannenstiel incision is made, and the surgical field is maintained with the use of a wound retractor (Alexis^®^). After wiping the surface of the tumor located just under the wound with gauze, the rough side of a sterilized surgical sheet (ELS-102S) is attached to the cyst using quick-drying glue (Aron Alpha A <Sankyo>^®^) with careful attention not to spread the glue into the abdominal cavity (Fig. 1a). (Quick-drying glue cannot work when applied to the vinyl coating side of the sheet.) After the sheet is glued tightly to the tumor, requiring several seconds, the cyst is punctured with a suction tube, and the cyst fluid is aspirated as extensively as possible (Fig. 1b). The cyst shrinks, and the tumor is then brought outside of the body, while tightly clamping the punctured site to prevent spillage of cyst fluid remaining in the tumor. This procedure is applicable to multiocular cyst by puncturing septums successively in direct vision after part of the cyst is brought outside of the body. Finally, an ovarian cystectomy is performed, while safely preserving the healthy ovarian tissue on the affected side (Fig. 1c).

## Case reports

### Patient 1

**Fig. 2:**
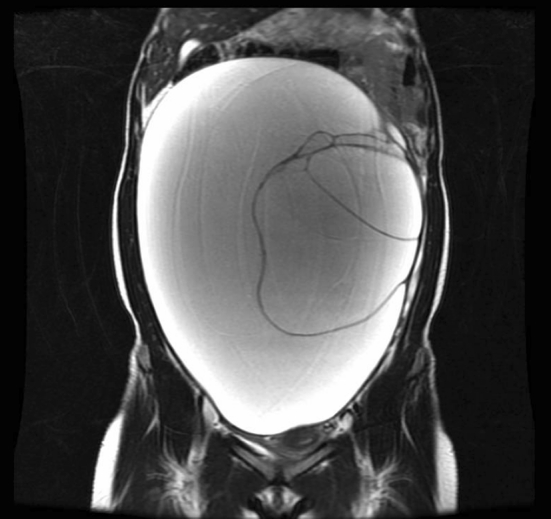
Patient 1: MRI (T2WI) showed a large multiocular ovarian cyst

**Fig. 3:**
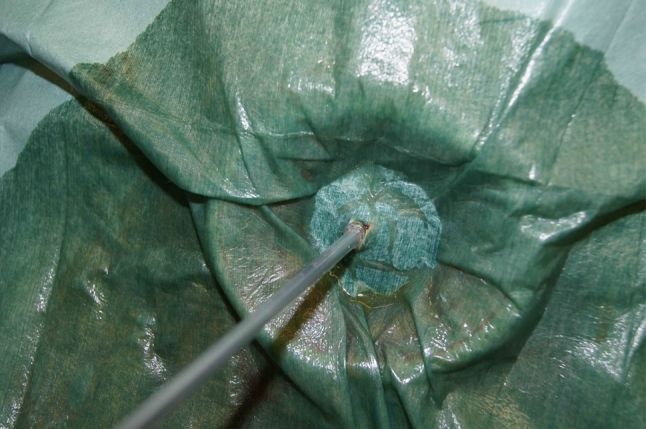
Patient 1: Puncturing the cyst using a S.A.N.D. balloon catheter after covering the lesion with sterilized surgical sheet (ELS-102S), applied using quick-drying glue (Aron Alpha A <Sankyo>^®^)

**Fig. 4:**
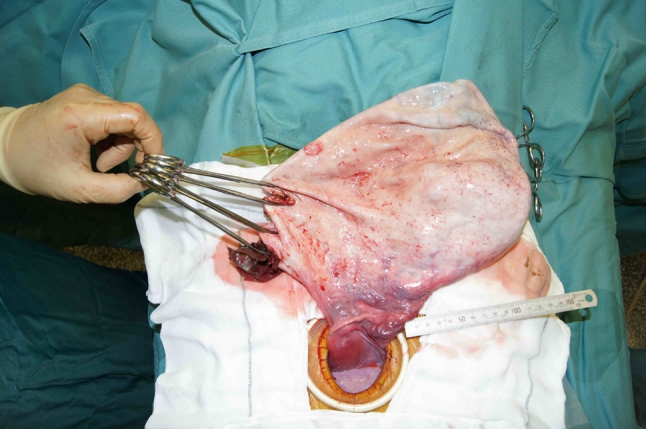
Patient 1: A large ovarian cyst after aspiration of the cyst fluid

A 15-year-old girl with a large abdominal mass was referred to our hospital. Physical examination showed a distended abdomen, but the patient had no complaints. Computed tomography (CT) and magnetic resonance imaging (MRI) revealed a 33 × 25 × 15 cm multiocular ovarian cystic tumor without calcification or fat tissue (Fig. 2). Neither ascites nor dissemination was evident. A serous cystadenoma or mucinous cystadenoma was suspected, and operation was performed. We made a 6 cm Pfannenstiel incision and punctured the cyst using a S.A.N.D. balloon catheter after covering the lesion with sterilized surgical sheet, applied using quick-drying glue (Fig. 3). After we aspirated about 7,200 ml of serous yellowish cyst fluid, we performed an ovarian cystectomy while preserving the healthy ovarian tissue, with no spillage of cyst fluid (Fig. 4). The histopathological diagnosis was a mucinous cystadenoma without malignancy including borderline lesions. The postoperative course was good, and the patient was discharged on the fourth postoperative day.

### Patient 2

A 5-year-old girl with abdominal pain was diagnosed with torsion of a 7.5 × 7.3 × 4.7 cm ovarian tumor on CT. Neither ascites nor dissemination was apparent. She was referred to our hospital and underwent emergency surgery. We made a 3 cm Pfannenstiel incision and punctured the cyst using a needle connected to a suction after covering the surface of the tumor with a sterilized surgical sheet, applied using quick-drying glue. After aspiration of about 140 ml of serous cyst fluid, we performed a cystectomy, preserving the healthy ovarian tissue without spillage of cyst fluid. The histopathological diagnosis was a mature cystic teratoma. She was discharged without any complications on the fourth postoperative day.

### Patient 3

**Fig. 5:**
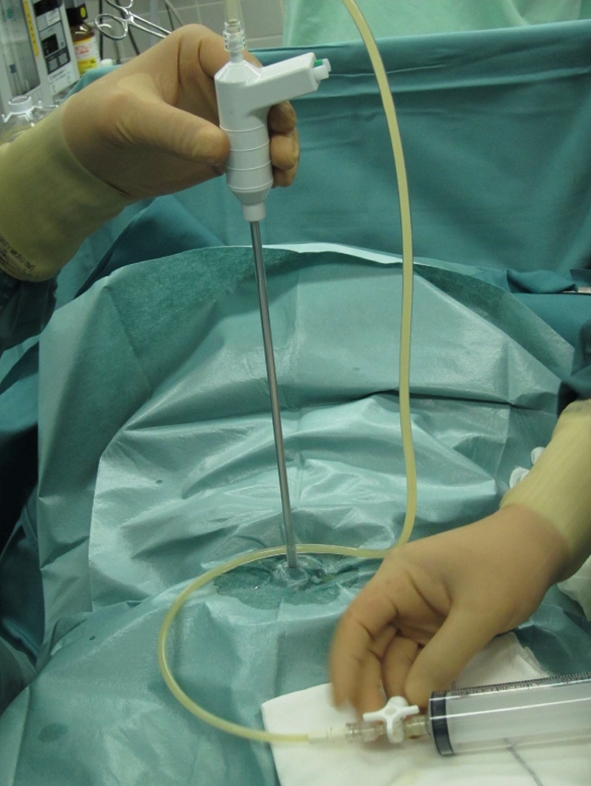
Patient 3: After covering the cystic tumor with a sterilized surgical sheet (ELS-102S), applied using quick-drying glue, the cyst was punctured with a S.A.N.D. balloon catheter. About 940 ml of yellowish, turbid cyst fluid was aspirated, with no spillage

A 14-year-old girl with inferior abdominal pain was found to have bilateral multiocular ovarian tumors (right: 6.5 × 4.8 × 4.3 cm, left: 17 × 13 × 9.0 cm) on CT and MRI. Physical examination showed a distended abdomen, but there were no complaints. Mature cystic teratomas were suspected, and operation was performed. We made a 6 cm Pfannenstiel incision and punctured the cyst using a S.A.N.D. balloon catheter after covering the surface of the left large cystic tumor with a sterilized surgical sheet, applied using quick-drying glue. After we aspirated about 940 ml of yellowish, turbid cyst fluid (Fig. 5), we performed a cystectomy. The healthy ovarian tissue was preserved. There was no spillage of cyst fluid. We subsequently performed a right ovarian cystectomy. The procedure was completed uneventfully. The histopathological diagnosis was a mature cystic teratoma. She was discharged without any complications on the fifth postoperative day.

### Patient 4

A 13-year-old girl with inferior right abdominal pain was found to have a 16 × 12 × 9.0 cm right multiocular ovarian tumor on CT. Mature cystic teratoma was suspected, and operation was performed. We made a 4.5 cm Pfannenstiel incision and punctured the cyst using a S.A.N.D. balloon catheter after covering the surface of the tumor with a sterilized surgical sheet, applied using quick-drying glue. After we aspirated about 1,000 ml of dark red, turbid cyst fluid, we performed a cystectomy. The healthy ovarian tissue was preserved. There was no spillage of cyst fluid. The procedure was completed uneventfully. The histopathological diagnosis was a mature cystic teratoma. She was discharged without any complications on the fifth postoperative day.

## Discussion

The treatment of choice for ovarian cystic tumors is generally surgical excision owing to the risks of ovarian torsion, spontaneous rupture, and malignancy [7]. Traditionally, open laparotomy was the standard procedure for ovarian cystectomy.

However, laparoscopic procedures have recently become more popular, similar to other surgical specialties. Many reports have documented advantages of laparoscopic procedures for ovarian cystectomy, such as enhanced visualization of the entire pelvis, reduced need for analgesia, shorter hospital stay, early recovery and resumption of routine activities, and improved cosmetic outcomes [6–11]. But the rate of cyst rupture during laparoscopic surgery has been reported to range between 6 and 100 % [7, 9, 10, 12], and consequently, laparoscopic surgery can be associated with increased risks of chemical peritonitis, gliomatosis peritonei, pseudomyxoma peritonei, tumor recurrence, and dissemination of malignant cells caused by spillage of ovarian cyst fluid during surgery [1–6]. There are some reports that gliomatosis peritonei derived from the component of ovarian teratomas following rupture of their capsules [1, 2]. Recently, it is said that pseudomyxoma peritonei does not correlate to mucinous ovarian tumors [13], but there were some cases where it was associated with intestinal mucinous cystadenomas of the borderline malignancy [3]. In addition, regarding the recurrence, there was a significantly higher rate of intraoperative cyst rupture in the cases of cyst recurrence, compared with those with no recurrence in the study of the benign ovarian mucinous cystadenoma [4]. Thus, complete prevention from spillage of cyst fluid is necessary for cystic ovarian tumors.

In response to such problems, many studies have reported on ways to prevent spillage of the cyst fluid during open [14, 15] as well as laparoscopic abdominal surgery [5, 6, 16]. However, whether all procedures are completely reliable has been questioned because spillage of some cyst fluid is unavoidable when surgeons directly puncture the cysts. The spillage of large amounts of cyst fluid in patients who already have spillage becomes a particularly high risk, especially in laparoscopic operations. Therefore, much safer methods are needed, particularly in pediatric surgery.

We developed a new technique to completely prevent spillage of cyst fluid. Quick-drying glue used in our procedure is made from cyanoacrylate which is widely used in surgical field [17–19]. In addition, quick-drying glue has never dropped into abdominal cavity, because requirement of glue is not so much. In all four patients described above, there was no spillage of cyst fluid, and the healthy ovarian tissue could be preserved without any complications (Table 1). Inexperience on the part of surgeons has been linked to a high risk of cyst rupture during laparoscopic procedures [12]. On the other hand, we believe that our technique is much safer and more feasible for all surgeons not only for conventional but also for laparoscopic surgery. As for cosmetic results, a small single incision is better than laparoscopic surgery because the latter requires another incision through which the excised cyst is exteriorized. Moreover, additional costs associated with our technique are considered minimal.

**Table 1:** Clinical characteristics of four patients in our experience

Case	Side	Tumor size (cm)	Operation	Skin incision (cm)	Septum	Cyst fluid (ml)	Spillage	Diagnosis
1	Left	33 × 25 × 15	Cystectomy	6	+	7,200	−	MC
2	Left	7.5 × 7.3 × 4.7	Cystectomy	3	−	140	−	MCT
3	Left Right	17 × 13 × 9.0 6.5 × 4.8 × 4.3	Cystectomy Cystectomy	6^a^	+ +	9,400	− −	MCT MCT
4	Right	16 × 12 × 9.0	Cystectomy	4.5	+	1,000	−	MCT

*MC* mucinous cystadenoma, *MCT* mature cystic teratoma

^a^Larger incision was needed because bilateral tumors existed

In patients with large cystic ovarian tumors, puncturing the cyst after it is covered with a sterilized surgical sheet applied using quick-drying glue not only prevents spillage of cyst fluid into the abdominal cavity, but also has good cosmetic results. To our knowledge, this is the first report to describe the use of both a suction tube such as a S.A.N.D. balloon catheter and quick-drying glue for the treatment of abdominal cystic tumors. Our experience suggests that our technique is a safe and reliable procedure that can be used by all surgeons to prevent spillage of cyst fluid in patients undergoing surgery for cystic ovarian tumors.
